# Exeunt

**Published:** 1892-03-12

**Authors:** 


### PASSING TOPICS.

### EXEUNT.

The accidental juxtaposition in an evening paper of paragraphs headed "London Hospital," and the "Hunter's Improvement Society," coupled with the appearance of Lord Midleton's name and a consequent and very natural reference to fogs, combined to induce an assumption upon our part, and possibly of some other readers, which it is disappointing to find was not warranted by the circumstances. As a matter of fact, the proximity of the items enumerated was merely fortuitous; they were entirely independent one of the other. The London Hospital remains pretty much *in statu quo*, and in another quarter there is no real improvement. So much the worse perhaps it might appear for peace and quietness and the welfare of a great institution. But there is an important change in the treatment which may lead to satisfactory results more speedily than has lately seemed probable.

The Governors have grown weary of parley and have had recourse at last to action. By an overwhelming majority they have passed a resolution expressing their unabated confidence in the administration of the Hospital, their warm appreciation of the services of the House Committee, and cordial and hearty thanks to their officers and the nursing staff. In that resolution, and in the unequivocal assertion by the Chairman that it has come "to be a case of who shall manage the Hospital," lies the hope of better things.

Reckoned by numbers or weight the malcontents have never been formidable, but they have been aggressors, and that has given them the advantage. It is, we believe, an old and unquestioned military axiom that to offer a defence which is all defence is to fight a losing battle. There must be counter-attack, if only to inspirit the defenders, and it is precisely because the Chairman and the Governors have at least turned boldly upon their assailants that we witness an utter collapse of the assault, and an apparently unconditional surrender to the demands of that discretion which we are assured is the better part of valour.

The proceedings of the small and noisy group which has interrupted business so long would never have become serious but for the supineness of the hospital authorities, and the latter have paid the penalty in grievous loss to the institution. It is not that the farce has been played out. There were almost infinite possibilities of mischief yet unexpended, but the arena has been swept clear of the sorry actors, and even their perception must now realise that there is a limit to patience, while the Chairman and his colleagues may well regret that they have not sooner put their foot down.

The rejoicings of the nursing staff at the result of the recent meeting are easily understood. Nurses as a body are sensible and capable people, aud if there be anything a capable and self-respecting person holds in contempt and aversion, it is a gratuitous endeavour on the part of unbidden and unwelcome advocates to represent him as a fatuous ass, contentedly munching the thistle at his feet, when if he only held his head a little higher he might feed upon corn.

Agricola.

